# Unraveling the pathways from alexithymia to work engagement among critical care nurses: the mediating effects of social support and organizational trust

**DOI:** 10.3389/fpsyg.2026.1942846

**Published:** 2026-09-23

**Authors:** Sameer A. Alkubati, Naif F. Alshammari, Abdulhafith Alharbi, Saleh Z. Alhetar, Galal F. Albani, Elham S. Moqbel, Ayman Mohamed El-Ashry, Hamdan M. Albaqawi, Wesam T. Almagharbeh, Habib Alrashedi, Bushra Alshammari, Layla Alshammari, Bandar S. Alsaif, Aziza Z. Ali

**Affiliations:** 1 Department of Medical-Surgical Nursing, College of Nursing, University of Hail, Hail, Saudi Arabia; 2 Department of Psychiatric and Mental Health Nursing, College of Nursing, University of Hail, Hail, Saudi Arabia; 3 Department of Maternal and Child Health, College of Nursing, University of Hail, Hail, Saudi Arabia; 4 Department of Nursing, College of Applied Medical Sciences, Jouf University, Al-Qurayyat, Saudi Arabia; 5 Medical Surgical Nursing Department, College of Nursing, University of Tabuk, Tabuk, Saudi Arabia; 6 Department of Public Health, College of Public Health and Health Informatics, University of Hail, Hail, Saudi Arabia; 7 Department of Nursing Administration, College of Nursing, University of Hail, Hail, Saudi Arabia

**Keywords:** alexithymia, critical care nurses, organizational trust, Saudi Arabia, social support, work engagement

## Abstract

**Background:**

Critical-care nurses (CCNs) are exposed to intense emotional demands that may impair work engagement, particularly in the presence of alexithymia. However, the mechanisms that link alexithymia to work engagement remain unclear. This study aimed to determine how organizational trust and social support mediate the relationship between alexithymia and work engagement among CCNs in Hail, Northwest Saudi Arabia.

**Methods:**

This cross-sectional correlational study involved 308 CCNs in Hail from August to November 2025. Data were collected using the Toronto Alexithymia Scale (TAS-20), Oslo Social Support Scale (OSSS-3), Organizational Trust Scale, and Utrecht Work Engagement Scale (UWES). Data were analyzed using AMOS 24.

**Results:**

More than half of the CCNs (52.3%) exhibited a high level of alexithymia, followed by moderate (36%) and low (11.7%) levels. Higher alexithymia levels were significantly associated with lower levels of social support (β = −0.229, *p =* 0.001), organizational trust (β = −0.286, *p =* 0.001), and work engagement (β = −0.287, *p =* 0.001). By contrast, work engagement was positively impacted by social support (β = 0.185, *p =* 0.002) and organizational trust (β = 0.236, *p =* 0.001). Alexithymia negatively impacted work engagement via social support (indirect effect = −0.044, 95% CI: −0.140 to −0.011, *p =* 0.014) and organizational trust (indirect effect = −0.070, 95% CI: −0.158 to −0.028, *p =* 0.001). Overall, alexithymia significantly decreased work engagement (β = −0.401, *p =* 0.001).

**Implications for practice:**

Enhancing organizational trust and strengthening social support may buffer the negative effects of alexithymia on work engagement. Interventions such as leadership development, peer support programs, and emotional skills training may help promote psychological well-being and sustained engagement among CCNs.

**Conclusion:**

Alexithymia is a significant psychological barrier to work engagement among CCNs. Organizational trust and social support can effectively mitigate this impact.

## 1 Introduction

Critical care nurses (CCNs) often work in complex clinical settings and care for patients with serious and changing conditions, including severe trauma and fatal incidents (Li L. et al., 2025). They face verbal and physical assault by patients and their families (Berger et al., 2024). In Saudi Arabia, the nursing workforce is highly diversified, with a large reliance on foreign nurses, who frequently face acculturative stress, language hurdles, and distance from their major support systems. Previous Saudi evidence indicates that CCNs experience substantial occupational and psychological pressure (Alshammari et al., 2024). According to a scoping analysis, intrapersonal, interpersonal, and organizational aspects are significant contributors to the well-being of Saudi Arabian CCNs who reported moderate to high levels of burnout (Alzailai et al., 2021). They are exposed to heavy workloads, retention pressures, language restrictions, and cultural adjustment demands (Alsadaan et al., 2021). A recent Saudi meta-analysis found that 61.8% of nurses experienced workplace violence, with the highest exposure rates in intensive care and afternoon-shift units (Abdelmalik et al., 2025). Such contextual pressures can strain emotional resources and make it difficult to maintain a level of work engagement. These situations place substantial psychological and mental stress on them, impacting their health and ability to remain adaptable and committed to their clinical duties (Alkubati et al., 2025).

As a key factor in sustaining professional health and achievement, work engagement describes an individual’s passion, enthusiasm, and devotion to their profession. It is defined as a positive, rewarding, and professionally related state of mentality, with three dimensions: vigor, dedication, and absorption (Taris, 2023). Employees who are engaged, interested in, dedicated to, and compassionate about their work and who believe that their efforts are valuable can improve organizational performance (Nabhan and Munajat, 2023). They also tend to connect their well-being, values, and privileges with the organization’s successes, which is crucial to the performance and achievements of both employees and their organizations. In a study of 900 nurses in the United States, work engagement was positively correlated with job satisfaction and perceived QoC (Wei et al., 2023).

CCNs’ well-being, performance, and work engagement of CCNs are influenced by alexithymia, a personality trait associated with emotional control issues (van der Velde et al., 2013). The hallmark of alexithymia is the inability to identify feelings, an externally oriented way of thinking, an inadequate imagining of life, and an inability to distinguish between feelings of emotional excitement and physical sensations (Jakobson and Rigby, 2021). Alexithymia is a risk factor for several psychiatric and neurological conditions (van der Velde et al., 2013), affects approximately 10% of the general population (Jakobson and Rigby, 2021; Mattila et al., 2010). In China, 43.7% of nurses working in intensive care units (ICU) were found to have alexithymia (Nan et al., 2023). Alexithymia among nurses can lead to various negative consequences, including diminished stress control, communication with others, and integration into society (Korkmaz et al., 2020; Nan et al., 2023). It can also predict mental health issues such as stress, depressive disorders, compassion fatigue, and diminished work engagement (Korkmaz et al., 2020; Li Y. et al., 2025; Zhang et al., 2021).

Alexithymia may reduce work engagement through interrelated psychological and interpersonal processes. Nurses who struggle to identify and understand emotions may be unable to properly recognize, interpret, or convey their emotional requirements. This may impede interpersonal communication and the formation of supportive connections between coworkers and other members of the healthcare team (Dalokay and Aydin, 2023). According to more recent research among nurses (Li Y. et al., 2025). This emphasizes the significance of interpersonal and occupational processes in explaining the effects of alexithymia. Therefore, nurses may be less able to seek out, perceive, or make efficient use of interpersonal and organizational resources that are accessible if they have trouble recognizing and expressing their emotions (Ejheisheh et al., 2025). Nurses’ ability to remain vigorous, committed, and absorbed in their work may be hampered by their limited access to these resources, which would eventually undermine work engagement (Alharbi et al., 2025).

Many strategies such as social support and organizational trust may lessen the negative impact of alexithymia on work engagement. Social support refers to the actual or perceived physical and emotional comfort offered by friends, family, partners, and coworkers (Acoba, 2024), which reduces burnout and psychological suffering among CCNs. Due to difficulties in communicating their psychological needs, alexithymic people may find it difficult to seek out or use social assistance, but a supportive environment is still crucial. Social support fosters a sense of belonging that keeps workers inspired and committed to their work, enhances emotional regulation, and enables problem-solving in difficult circumstances (Garmendia et al., 2023). In addition, it has a negative relationship with alexithymia (Pei et al., 2021).

Organizational trust describes how employees subjectively perceive their workplace, particularly how welcoming and safe they believe it is (Zhang et al., 2024). A negative correlation was found between alexithymia and increased work engagement. Perceptions of fairness, psychological safety, and willingness to work may all be affected by employees’ trust in their organization and its leaders, which can improve their job attitudes and adaptive behaviors. Organizational trust is positively connected with nurses’ job satisfaction and team-based practices (Huang et al., 2024), which improves work engagement and reduces alexithymia.

### 1.1 Theoretical framework

The Job Demands-Resources (JD-R) model (Demerouti et al., 2001) and Conservation of Resources (COR) theory (Hobfoll, 2011) served as the foundation for this study’s conceptual framework. The JD-R model primarily explains the occupational mechanism through which job resources influence employee motivation and engagement, whereas COR theory provides a broader explanation of how individuals respond to resource loss, protect existing resources, and invest additional resources to prevent further loss and facilitate recovery.

Within this coupled framework, alexithymia is defined as a profound internal psychological vulnerability that limits nurses’ capacity to identify, understand, and control their emotions. Difficulties in identifying, comprehending, and controlling emotions can deplete psychological resources and impair nurses’ capacity to recognize, seek, mobilize, and effectively use interpersonal and organizational resources. According to COR theory, such difficulties can initiate or exacerbate a resource-loss process; nurses who struggle to process and communicate emotional experiences may have more difficulty maintaining supportive interpersonal relationships and developing a sense of psychological security and trust in their workplace (Hobfoll, 2011). As a result, restricted access to or the use of valued resources may reduce the psychological energy available for long-term work involvement.

By highlighting the function of organizational trust and social support as job resources in a demanding critical care setting, the JD-R model enhances this viewpoint of resource loss. While organizational trust is an organizational resource linked to psychological safety, justice, and confidence in the workplace and its leadership, social support offers interpersonal and emotional resources. The JD-R model states that these resources can lessen the negative impacts of demanding work environments and promote a motivational process that results in higher levels of engagement at work (Demerouti et al., 2001). Therefore, the JD-R model explains how accessible job resources may mitigate these negative impacts and maintain nurses’ motivation and engagement, whereas the COR theory explains why alexithymia may be linked to the depletion or impaired utilization of psychological and social resources.

Because each represents a theoretically unique resource pathway by which alexithymia may be linked to work engagement, social support and organizational trust were proposed as parallel mediators rather than sequential mediators. While organizational trust functions through relational and organizational security, social support functions primarily through the supply of interpersonal and emotional resources. Using the JD-R and COR models, this study methodically examined how alexithymia undermines organizational trust and social support required for optimal psychological functioning and work engagement in an extremely demanding critical care setting.

Although earlier research has shown that alexithymia is linked to negative psychological and professional outcomes for nurses, evidence is still dispersed. For instance, alexithymia has been associated with psychological distress, burnout, social support, and other professional outcomes in Chinese studies on critical care and emergency nurses (Nan et al., 2023; Pei et al., 2021). Similar links between alexithymia and nurses’ communication abilities, mental health, burnout, anger, and somatization have been found in Turkish research (Dalokay and Aydin, 2023; Korkmaz et al., 2020).

Evidence from the Middle East suggests that psychological and organizational resources are major predictors of nurses’ work engagement. Emotional intelligence, resilience, and psychological empowerment have been linked to higher levels of professional engagement among Saudi critical care nurses (Abualruz et al., 2024). More recently, Saudi research has identified organizational support and psychological capital as critical resources related to nurses’ professional engagement (Alotaibi, 2026; Alqhtani et al., 2026). However, these studies have examined psychological or organizational resources independently, rather than alexithymia, as a predictor of job engagement via the simultaneous mediating functions of organizational trust and social support.

The regional evidence gap is not a lack of research on nurses’ psychological resources in the Middle East, but there is a lack of an integrated model that investigates how emotion-related vulnerabilities, such as alexithymia, influence work engagement via organizational and interpersonal resources. These limited regional data are especially important in critical care nursing, where nurses face ongoing emotional pressures, high-acuity clinical circumstances, and complicated interpersonal and organizational environments. Thus, investigating these associations among critical care nurses in Saudi Arabia provides regionally relevant findings while expanding past research exclusively beyond East Asian contexts. Therefore, this study aimed to identify the roles of CCNs in Hail City, Northwest Saudi Arabia. The hypotheses for this study are as follows:

Hypothesis 1: There is a negative relationship between alexithymia and work engagement among CCNs.

Hypothesis 2: There is a negative relationship between alexithymia and organizational trust among CCNs.

Hypothesis 3: There is a negative relationship between alexithymia and social support among CCNs.

Hypothesis 4: There is a positive relationship between organizational trust and work engagement among CCNs.

Hypothesis 5: There is a positive relationship between social support and work engagement among CCNs.

Hypothesis 6: CCNs’ organizational trust and social support mediate the relationship between alexithymia and work engagement.

## 2 Materials and methods

### 2.1 Design, participants and settings

From August to November 2025, a cross-sectional correlational study, in accordance with the STROBE guidelines, was conducted using a convenience sample of Saudi and non-Saudi CCNs at public hospitals in Hail City. Nurses were excluded if they had less than one year of critical care experience, performed administrative or auxiliary support roles, or declined to participate in the study. A minimum sample size of 266 was determined using G*Power, version 3.1.9.4 (Heinrich Heine University, Düsseldorf, Germany). This calculation was based on an initial power analysis using a bivariate correlation model and a one-tailed test with an estimated small effect size of 0.20, power of 0.95, and significance level of 0.05. However, because this study utilized a multiple-mediation structural equation model (SEM), which requires more rigorous sampling, sample size adequacy was further verified using SEM-specific criteria. According to the established guidelines for path analysis and SEM, a minimum sample size of 200 is recommended to ensure model stability and adequate statistical power. The structural path model in this study estimated a limited number of parameters across the four primary constructs. To increase the sensitivity and ensure that these SEM assumptions were robustly met, 350 questionnaires were distributed. Of these, 308 were completed and returned (response rate of 88%), yielding excellent statistical power for parallel mediation path analysis.

### 2.2 Study instruments

A questionnaire with five sections was used to collect the data. The original English versions of all the measurement scales were administered. English is the official working language in participating hospitals, and nurses routinely use it for clinical communication, documentation, and professional practice. The first section covered CCNs’ demographics and practices, including age, sex, marital status, years of experience, shift pattern, and additional work outside the hospital. The second section consisted of the Toronto Alexithymia Scale (TAS-20) created by Bagby et al. (1994). Using a 5-point Likert scale, with “1” for ‘strongly disagree’ and “5” for ‘strongly agree,’ this scale measures alexithymia traits in clinical and non-clinical settings. The tool’s overall score ranges from 20 to 100, with higher scores representing a greater level of alexithymia. The degree of alexithymia was classified as low (≤51), moderate (52–60), or high (≥61) based on the TAS-20 score cutoff values suggested by Taylor et al. (1997). Permission to use TAS-20 was formally obtained from R. Michael Bagby, a copyright holder (Bagby et al., 1994). The reliability of the TAS-20 in assessing alexithymia has been demonstrated across multiple languages and populations (El Abiddine et al., 2017; Palma-Álvarez et al., 2022), and its reliability was verified by a Cronbach’s α of 0.771 (Arenliu et al., 2021). With a Cronbach’s α of 0.853, this study similarly concluded that the tool is reliable.

The third section included three questions to measure the level of social support using the Oslo Social Support Scale (OSSS-3) [26]. The overall score ranged from 3 to 14 points, with higher scores indicating greater social support. Based on these scores, Kaddourah et al. (2018) and Zhu et al. (Zhu et al., 2023) divided social support into three categories: weak (3–8), moderate (9–11), and strong (12–14). The reliability of the OSSS-3 was excellent, with a Cronbach’s α of 0.920 (Alshammari et al., 2023). With a Cronbach’s α of 0.809, it was determined to be reliable in this study.

In the fourth section, CCNs’ organizational trust was measured using the Organizational Trust Scale created in 2006 (Chen, 2006). This scale includes 13 items to measure three dimensions: five for trust in the organization, four for trust in leadership, and four for trust in colleagues. A 5-point Likert scale was used to rank the responses, with “1” representing ‘strongly disagree’ and “5” denoting ‘strongly agree. ‘ Higher scores indicate better organizational trust, which varies from 13 to 65 (Zhang et al., 2024; Zheng et al., 2024). A previous study verified its reliability with a Cronbach’s α of 0.968 (Zhang et al., 2024). The reliability established in this study was excellent, with a Cronbach’s α of 0.935.

The Utrecht Work Engagement Scale (UWES), comprising 17 items developed by Schaufeli et al. in 2002 (Schaufeli et al., 2002) was used in the fifth section to measure work engagement. The three subscales of this scale – six items for vigor, five for dedication, and six for absorption – represent the fundamental characteristics of engagement. These items were scored on a 7-point Likert scale. The overall score ranges from 0 to 102, with higher scores indicating higher workplace involvement. With a Cronbach’s α of 0.896, the reliability of the UWES tool has been previously verified (Chaudhary et al., 2012). The reliability established in this study was excellent, with a Cronbach’s α of 0.921.

### 2.3 Data collection and ethical considerations

The Institutional Review Board of the University of Ha’il approved this study (Ethical Approval No: H-2025-738), and the ethical standards of the Declaration of Helsinki were followed. To ensure comprehension and clarity, the researchers provided written and verbal instructions before administering the questionnaires. Participants were briefed on the purpose of the study and informed of their freedom to discontinue participation without consequences. Participants’ anonymity and confidentiality were maintained.

### 2.4 Data analysis

Data were analyzed using IBM SPSS Statistics version 27 (IBM Corp., Armonk, NY, USA). Prior to the analysis, the dataset was examined for missing data, completeness, outliers, distributional assumptions, and univariate and multivariate normality. Acceptable skewness and kurtosis values, ranging from −0.490 to +1.005, were used to confirm univariate normality. In addition, multivariate normality was confirmed, with a Mardia kurtosis coefficient of 1.468 and a critical ratio of 1.859. As the multivariate critical ratio was below the conventional threshold of 1.96, the assumption of multivariate normality was not significantly violated (Martynova et al., 2018). These normality checks supported the suitability of the parametric tests and structural equation modeling for subsequent analyses. Accordingly, Pearson’s correlation coefficient was used to test the correlations between the study variables. Since all data were collected via self-administered questionnaires at a single time point, both procedural and statistical methods were employed to minimize and assess common method bias. Participants were informed that there were no correct or incorrect answers, that participation was voluntary, and that their responses would remain anonymous and confidential to lessen evaluation anxiety and social desirability bias. We also used Harman’s single-factor test to statistically assess common technique bias. According to exploratory factor analysis, the first unrotated factor accounted for 39.906% of the total variance. This is less than the suggested threshold of 50%, suggesting that common method bias is unlikely to have significantly affected the conclusions (Podsakoff et al., 2003).

The mediating roles of organizational trust and social support in the relationship between alexithymia and work engagement among CCNs were investigated using a bootstrap approach (2000 repetitions, 95% bias-corrected confidence interval) and path analysis using AMOS 24.0. The fit of the model was assessed using the following indices: root mean square error of approximation (RMSEA) < 0.08, comparative fit index (CFI) > 0.90, normed fit index (NFI) > 0.90, goodness-of-fit index (GFI) > 0.90, Tucker–Lewis index (TLI) > 0.90, and chi-square to degrees of freedom ratio (χ^2;^/df) < 3 (Martynova et al., 2018). Statistical significance was set at *p <* 0.05.

## 3 Results

### 3.1 Characteristics of participating nurses

The mean age of CCNs included in the study was 32.3 ± 7.3 years, with over half of the participants (52.6%) aged 31 years or younger. The sex distribution was almost even, with 51.3% female and 48.7% male participants. Most nurses were married (51%), followed by single (40.3%) and divorced or widowed (8.7%). On average, participants had 7.5 ± 5.6 years of work experience, with more than half having 6 years or less of experience (54.9%). Most participants had a bachelor’s degree (52.9%), followed by a diploma (36.4%) and a master’s degree (10.7%). Concerning shift patterns, approximately two-thirds of the nurses reported irregular schedules, while one-third reported regular schedules. Most participants reported dissatisfaction with their work (40.6%), followed by those who were partially satisfied (30.2%) and satisfied (29.2%). On the other hand, the majority of the participants (71.8%) did not engage in extra work outside their main duties (Table 1).

**TABLE 1:** Sociodemographic and work-related characteristics of study participants (*N* = 308).

Characteristics	*n*	(%)
Age (years)		
Mean ± SD: 32.3 ± 7.3		
≤31	162	(52.6)
>31	146	(47.4)
Gender		
Male	150	(48.7)
Female	158	(51.3)
Nationality		
Saudi	178	(57.8)
Non-Saudi	130	(42.2)
Marital status		
Single	124	(40.3)
Married	157	(51.0)
Divorced/widowed	27	(8.7)
Length of experience (years)		
Mean ± SD: 7.5 ± 5.6		
≤6	169	(54.9)
>6	139	(45.1)
Level of education		
Diploma	112	(36.4)
Bachelor	163	(52.9)
Master	33	(10.7)
Shift pattern		
Regular schedule	111	(36.0)
Irregular schedule	197	(64.0)
Satisfaction with working		
Yes	90	(29.2)
No	125	(40.6)
Partially	93	(30.2)
Extra outside work		
Yes	87	(28.2)
No	221	(71.8)

SD, standard deviation.

### 3.2 CCNs’ levels of alexithymia, organizational trust, social support and work engagement

Table 2 shows that CCNs exhibited a high level of alexithymia, as indicated by a mean alexithymia score of 62.95 ± 11.82 and a mean percentage score (MPS) of 53.68%. Organizational trust was comparatively lower, with a mean score of 38.68 ± 3.93 and an MPS of 49.38%, suggesting a moderate to low level of trust in the organization. Nurses also showed moderate perceived social support, as indicated by a mean score of 8.31 ± 2.36 and an MPS of 48.27%. In contrast, nurses demonstrated a comparatively higher level of work engagement, with a mean score of 68.89 ± 18.98 and an MPS of 67.54%. A supplementary subgroup analysis comparing these variables between Saudi national (n = 178) and non-Saudi expatriate nurses (n = 130) is detailed in Supplementary Table 1.

**TABLE 2:** Relationship between alexithymia, organizational trust, social support and work engagement among CCNs (*N* = 308).

Variables	Mean ± SD	MPS	(r)/*p*-value	1	2	3	4
1. Alexithymia	*62.95 ± 11.82*	53.68	*r*	1			
			*P*				
2. Organizational trust	*38.68 ± 3.93*	49.38	*r*	−0.286**	1		
			*P*	<0.001			
3. Social support	*8.31 ± 2.36*	48.27	*r*	−0.229**	0.140*	1	
			*P*	<0.001	0.014		
4. Work engagement	*68.89 ± 18.98*	67.54	*r*	−0.396**	0.343**	0.283**	1
			*P*	<0.001	<0.001	<0.001	

CCNs, critical care nurses; SD, standard deviation; MPS, Mean percentage score *p < 0.05; **p < 0.01.

Alexithymia was negatively correlated with organizational trust (r = −0.286, *p <* 0.001), social support (r = −0.229, *p <* 0.001), and work engagement (r = -0.396, *p <* 0.001). By contrast, organizational trust was positively correlated with social support (r = 0.140, *p =* 0.014) and work engagement (r = 0.343, *p <* 0.001). Similarly, social support was positively correlated with work engagement (r = 0.283, *p <* 0.001) (Table 2).

Figure 1 illustrates that more than half of the CCNs had a high alexithymia level (52.3%), followed by moderate (36%) and low (11.7%) levels.

**FIGURE 1:**
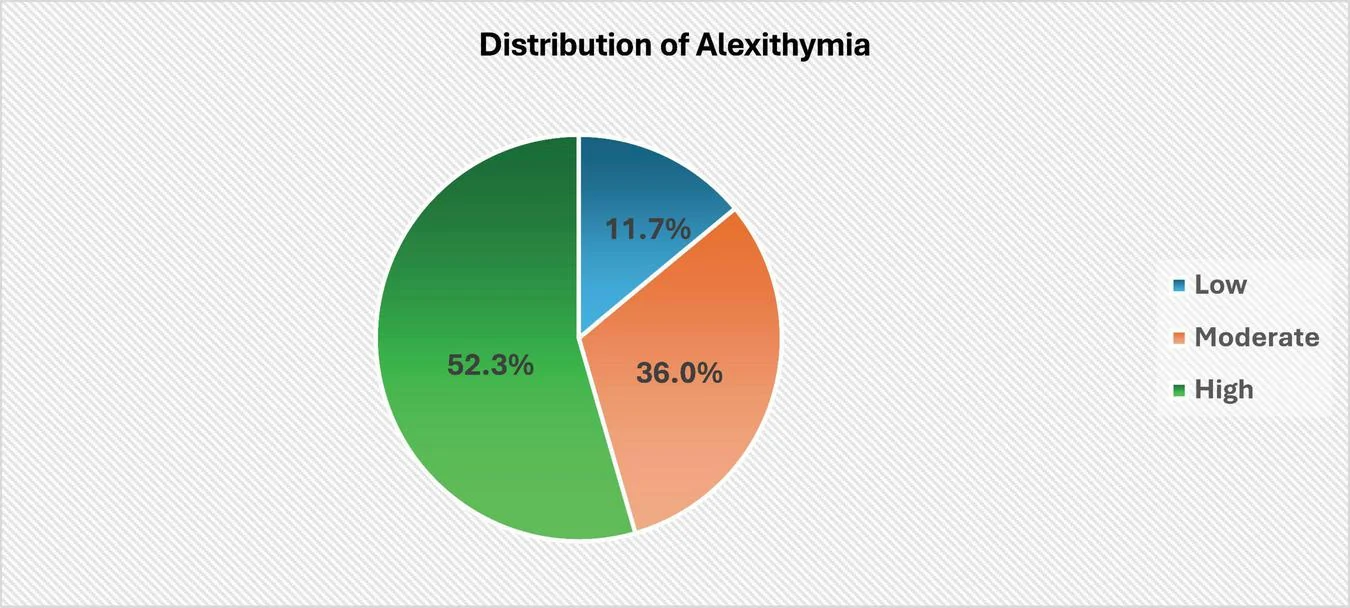
Distribution of alexithymia levels among CCNs.

### 3.3 Organizational trust and social support mediating effects on the relationship between alexithymia and work engagement

The path analysis model demonstrated an acceptable to excellent fit to the data, as all fit indices met the recommended cutoff values (χ^2^/df = 1.954, TLI = 0.954, CFI = 0.992, NFI = 0.985, GFI = 0.997, RMSEA = 0.056, *p =* 0.162) (Table 3).

**TABLE 3:** Model fit indices for the path analysis model.

Model	χ ^2^/df	TLI	CFI	NFI	GFI	RMSEA (*p*-value)
Acceptable threshold	≤3.00	≥0.90	≥0.90	≥0.90	≥0.90	≤0.08 (>0.05)
Emerging model	1.954	0.954	0.992	0.985	0.997	0.056 (0.162)

χ^2^, Chi-square; df, degrees of freedom; TLI, Tucker–Lewis index; CFI, comparative fit index; NFI, normed fit index; GFI, goodness of fit index; RMSEA, root mean square error of approximation. All indices indicated an acceptable-to-excellent model fit based on the recommended cut-off values.

Path analysis revealed statistically significant negative associations between alexithymia and both mediators (Figure 2; Table 4). Higher alexithymia was associated with lower social support (β = −0.229, *p =* 0.001), lower organizational trust (β = −0.286, *p =* 0.001), and lower work engagement (β = −0.287, *p =* 0.001). Work engagement was positively associated with social support (Î^2^ = 0.185, *p =* 0.002) and organizational trust (Î^2^ = 0.236, *p =* 0.001). The bootstrapped indirect association through social support was −0.044 (95% CI: −0.140 to −0.011, *p =* 0.014), and the indirect association through organizational trust was −0.070 (95% CI: −0.158 to −0.028, *p =* 0.001). The total association between alexithymia and work engagement was −0.401 (*p =* 0.001). These coefficients describe cross-sectional statistical paths and do not establish a temporal mediation.

**FIGURE 2:**
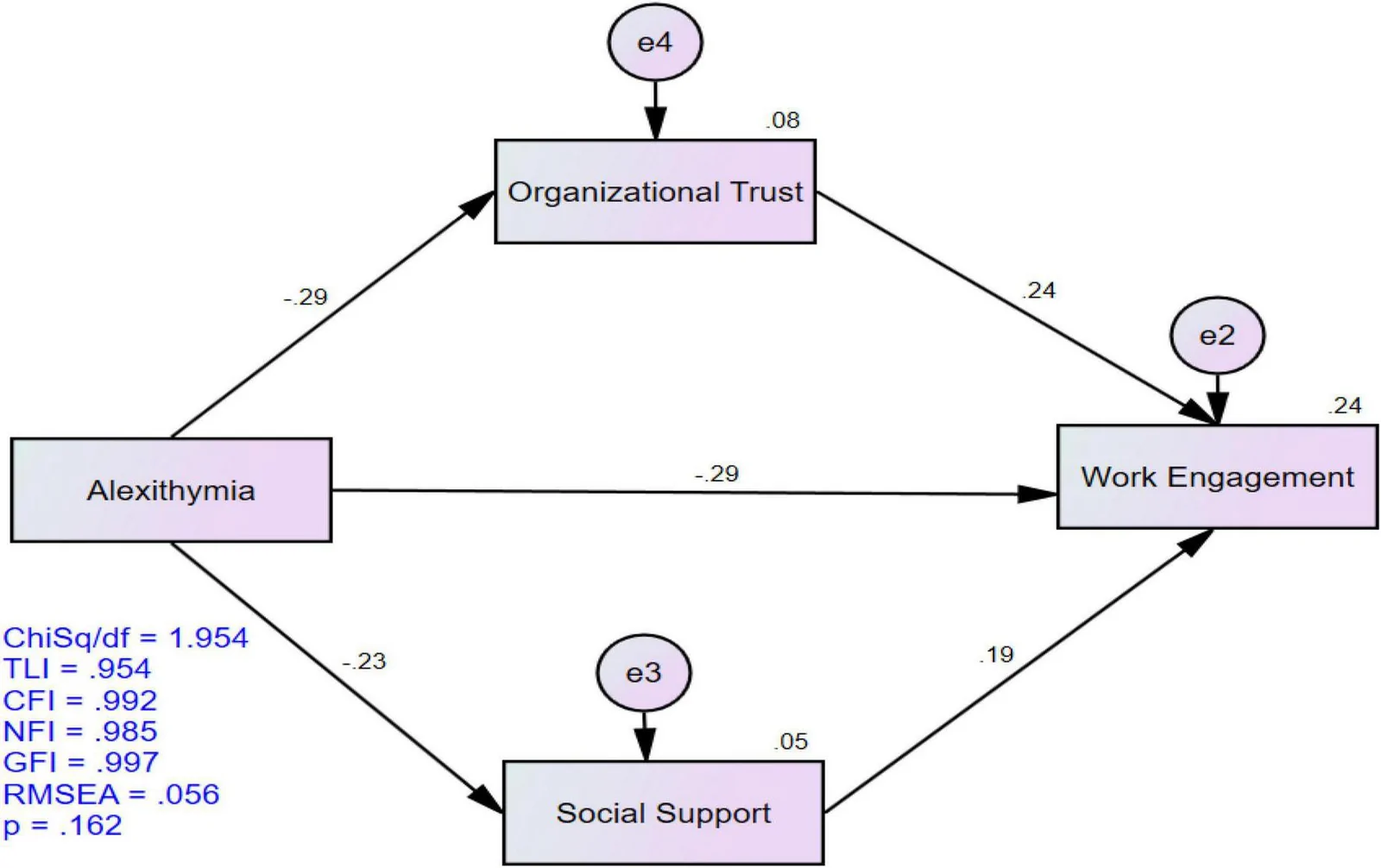
The mediating roles of organizational trust and social support in the relationship between alexithymia and work engagement.

**TABLE 4:** Path coefficients for the associations among alexithymia, social support, organizational trust, and work engagement among CCNs.

Path	Effect size	95% CI		*p*-value
		**(LLCI)**	**(ULCI)**	
Indirect effect				
Alexithymia > Social support > Work engagement	−0.044	−0.140	−0.011	0.014
Alexithymia > Organizational trust > Work engagement	−0.070	−0.158	−0.028	0.001
Direct effect				
Alexithymia > Social support	−0.229	−0.341	−0.109	0.001
Alexithymia > Organizational trust	−0.286	−0.366	−0.199	0.001
Social support > Work engagement	0.185	0.073	0.296	0.002
Organizational trust > Work engagement	0.236	0.142	0.323	0.001
Alexithymia > Work engagement	−0.287	−0.386	−0.186	0.001
Total effect				
Alexithymia > Work engagement	−0.401	−0.489	−0.298	0.001

Decomposition of total association: The indirect association through social support represented 11.0% of the absolute total association (0.044/0.401), and the indirect association through organizational trust represented 17.5% (0.070/0.401). Together, these two indirect paths represented 28.4% of the total association, leaving 71.6% as the direct pathway in this model. Of the combined indirect associations (0.114), organizational trust accounted for 61.4% and social support accounted for 38.6%. LLCI, lower limit of confidence interval; ULCI, upper limit of confidence interval

## 4 Discussion

This is the first study to demonstrate the partial mediation of both social support and organizational trust in the alexithymia-engagement link among CCNs. The findings elucidate the psychological mechanisms underlying work engagement among frontline nurses. By showing how alexithymia negatively affects work engagement, this study validates Hypothesis 1. Additionally, positive correlations between work engagement and organizational trust/social support and negative correlations between alexithymia and these factors support the mediation hypotheses (H2–H6). Path analyses showed that organizational trust and social support partially mediated the relationship between alexithymia and work engagement.

More than half of the CCNs in this study exhibited a high level of alexithymia (52.3%), which notably exceeded the 43.7% prevalence previously reported among ICU nurses in China (Nan et al., 2023). This high detection rate can be linked to specific contextual and demographic pressures of the Saudi healthcare system. Specifically, the regional workforce is primarily reliant on expatriate professionals, who make up 42.2% of the current sample and commonly experience extreme cultural stress, language hurdles, and separation from their primary familial support networks. Furthermore, the combination of difficult working conditions, such as unpredictable shift schedules indicated by 64% of the participants, severely limits opportunities for proper rest and emotional recovery. Rotating and irregular shifts might disturb sleep, recovery, emotional control, and the possibility of consistent social connections, potentially increasing psychological susceptibility. Third, workplace violence is a major occupational stressor in the Saudi healthcare setting. A recent systematic review and meta-analysis found a pooled prevalence of workplace violence of 61.8% among Saudi nurses and 68.1% among ICU nurses, showing that exposure to threatening or emotionally stressful interactions is a major problem in critical care settings (Abdelmalik et al., 2025). Consequently, this high-pressure local environment is likely to worsen emotional tiredness and dysregulation, leading to a higher prevalence of alexithymia than in more culturally and demographically homogeneous nursing groups. These explanations are based on context and not on mediators that have been tested; future research should directly assess stress related to migration, shift characteristics, violence exposure, and access to psychological services.

This study revealed a negative correlation between alexithymia and engagement. This finding aligns with the negative correlation between engagement and emotional disorders such as alexithymia among Chinese health professionals (Zhang et al., 2021). Additionally, among Chinese nurses, alexithymia is associated with emotional exhaustion and job satisfaction, which affects the engagement of some professionals (Li Y. et al., 2025). Emotional regulation issues may demand energy and absorption, two crucial components of work engagement. These elements support the concept of job demands and resources in relation to alexithymia, which is regarded as an internal factor impeding the efficient use of job resources (Demerouti et al., 2001).

Additionally, this study found a negative relationship between organizational trust and alexithymia. The importance of trust-building initiatives to foster feelings of safety, respect, and dedication in a work environment is highlighted by the fact that nurses with higher levels of alexithymia are less likely to trust the organization. Zheng et al. (2024) found that emotional difficulties contribute to decreased resilience and trust in an organizational framework (Zheng et al., 2024). By contrast, this study found that work engagement could be positively predicted by organizational trust, which is consistent with the findings of Huang et al. (2024) that trust promotes teamwork and job satisfaction (Huang et al., 2024). Organizational trust buffers the relationship between alexithymia and work engagement.

This study found a negative correlation between social support and alexithymia, indicating that CCNs with higher levels of alexithymia might perceive or receive less social support. Emphasizing social support could motivate health care practitioners to enhance the environment in which they support nurses’ well-being for increased nursing engagement. Garmendia et al. (2023) found that increased support from coworkers enhances motivation and reduces feelings of emotional fatigue in individuals with highly demanding roles. The indirect effect of social support on the alexithymia-engagement relationship indicates that external resources with strong emotional components are crucial for performance despite emotional challenges (Alshammari et al., 2025).

Although alexithymia directly influences work engagement, its effect is partially mediated by both organizational and social support. However, such effects can be mitigated by support at both the organizational and social levels. The indirect effects of social support and organizational trust in this study suggest a compensatory role. This study supports COR theory, which states that people dealing with emotional issues and work-related stress can cope with them more easily when they have access to resources (Hobfoll, 2011).

The magnitudes of the two indirect paths were different. The social support pathway (-0.044) accounted for 11.0% of the total association, while the organizational trust pathway (-0.070) accounted for 17.5%, making a total of 28.4%. Social support was associated with 38.6% of the indirect association, as opposed to organizational trust, which accounted for 61.4% of this indirect association. It is possible that engagement with trust is more pervasive than the social support measure, which is only global, because it is expressed in decisions, fairness, psychological safety, reporting, or team coordination on a day-to-day basis. This distinction implies that transparent leadership and fair systems should be prioritized, with peer and family support as complementary. The two indirect paths, however, had overlapping confidence intervals, and a formal contrast of the indirect effects was not reported; priority for interventions should not be based on point estimates alone.

The pattern is described by complementary JD-R and COR accounts. The JD-R defines trust and support as resources that are connected to the motivational process of engagement, and COR helps to understand why difficulties in emotion identification and emotion support-seeking could be related to the loss or underuse of those resources. The other pathway (71.6% of the total association) is also direct and suggests that other mechanisms (emotional exhaustion and burnout, depressive symptoms, workload, and leadership climate) should be explored and not used as full explanations of the association between trust and support.

### 4.1 Limitations

This study had a few limitations. First, this was a cross-sectional study, and causality could not be established. Path analysis models suggest mediators, but the correlation does not imply causation. Further longitudinal studies are required to confirm this hypothesis. In particular, because the constructs may have bidirectional relationships, there is a significant possibility of temporal confounding. For example, continuous experiences of low work engagement and occupational isolation may worsen nurses’ emotional dysregulation and alexithymic features over time, even though alexithymia may consume resources and impair work engagement. Future research must go beyond cross-sectional designs to separate these temporal dynamics and to determine clear causal pathways. Using a crossover panel design and at least three measurement time points (baseline, 6 months, and 12 months) throughout the 1-year follow-up period, we suggest a longitudinal follow-up study.

Second, self-reported questionnaires were used to measure all study variables simultaneously, which could have introduced social desirability, memory, and common method bias. The study was planned as a single-time-point cross-sectional survey; hence, no temporal separation or randomization of item order was used, despite the use of standardized and previously validated instruments. As a result, it is impossible to completely rule out common technique variance. Future longitudinal research should employ different data sources, randomize item presentations where appropriate, and use temporal separation between measures. Supervisor-rated performance or engagement, for instance, might be used to supplement job engagement, and objective metrics, such as staff retention, absenteeism, or turnover, could be included.

Third. Recruitment of nurses from Hail’s public hospitals may have limited the generalizability of the findings to private hospitals or other regions. Furthermore, there may be systematic differences among the non-responders. Although key demographic and work-related factors were measured, workload, nurse-to-patient ratio, workplace violence, burnout, depression/anxiety, leadership style, and unit climate may act as moderators or confounders in the relationships observed in this study.

Finally, although the instruments demonstrated acceptable internal consistency in the present sample, study-specific confirmatory factor analysis was not conducted. Therefore, the present study did not provide independent CFA-based evidence regarding the factor structure of the four instruments in Saudi critical care nurses. Future studies should employ CFA and, where appropriate, measurement-invariance analyses across language, nationality, and cultural groups to establish the structural validity and comparability of these measures further.

### 4.2 Implications for nursing & health policy

The results indicated that interventions should be adapted to the Saudi critical care context and applied at the individual, interpersonal, and organizational levels. At the individual level, hospitals may include short courses on emotional awareness and management in CCNs’ ongoing professional development programs. These courses concentrated on identifying, classifying, conveying, and effectively handling emotions. Psychological support should be offered in Arabic and English, with culturally appropriate methods for nurses from various cultural backgrounds, as the Saudi critical care workforce comprises both Saudi and foreign nurses. Trained mental health experts can lead to small-group counseling or emotional support sessions that utilize culturally appropriate communication techniques.

Nurse managers should prioritize initiatives to improve organizational trust and psychological safety, such as transparent communication, equitable workload and shift allocation, supportive leadership, and confidential mechanisms for reporting workplace problems or abuse. Regular staff feedback sessions and leadership check-ins can assist nurses in articulating their issues and gaining trust in organizational support. Mentoring and orientation that is sensitive to cultural differences may help foreign nurses adjust to their new employment. Thru open communication, equitable workload and shift distribution, encouraging leadership, and private reporting procedures, nurse managers can enhance psychological safety and organizational trust at the organizational level. In addition to strengthening organizational and interpersonal resources, these multidimensional approaches may encourage CCNs’ work engagement.

## 5 Conclusion

Alexithymia is a significant psychological barrier to work engagement among CCNs. Organizational trust and social support can effectively mitigate this impact. Implementing training programs to enhance emotional recognition and employ trust-building strategies may promote a resilient, engaged, and healthy nursing workforce.

## Data Availability

The raw data supporting the conclusions of this article will be made available by the authors, without undue reservation.
