# Supplementary material for: Unraveling the pathways from alexithymia to work engagement among critical care nurses: the mediating effects of social support and organizational trust
Source: Front Psychol. 2026 Sep 23;17:1942846. doi: 10.3389/fpsyg.2026.1942846 (PMC13642642; doi:10.3389/fpsyg.2026.1942846)
Supplement: Supplementary file 1 [file Table_1.docx]

**Supplementary Table S1: Comparison of alexithymia, organizational trust, social support, and work engagement between Saudi and non-Saudi critical care nurses**

| **Variables** | **Saudi CCNs (n = 178) Mean ± SD** | **Non-Saudi CCNs (n = 130) Mean ± SD** | **t-test** | **p-value** |
| --- | --- | --- | --- | --- |
| Alexithymia | 63.14±11.09 | 62.68±12.79 | .338 | .736 |
| Organizational trust | 38.88±4.07 | 38.40±3.74 | 1.074 | .284 |
| Social support | 8.34±2.49 | 8.25±2.16 | .326 | .745 |
| Work engagement | 69.77±18.18 | 67.69±20.04 | .951 | .343 |

Note: CCNs = critical care nurses; SD = standard deviation. Independent-samples t test
